# Role and promise of health policy and systems research in integrating rehabilitation into the health systems

**DOI:** 10.1186/s12961-024-01235-2

**Published:** 2024-10-09

**Authors:** Abdul Ghaffar, Abdulgafoor M. Bachani, Adnan A. Hyder, Alarcos Cieza, Aneel Bhangu, André Bussières, Diana C. Sanchez-Ramirez, Dorcas B. C. Gandhi, Jeanine Verbunt, Kumanan Rasanathan, Louise Gustafsson, Pierre Côté, Rajiv Reebye, Roger De la Cerna-Luna, Stefano Negrini, Walter R. Frontera, Sureshkumar Kamalakannan

**Affiliations:** 1 Department of Community Health Sciences, Aga Khan University, Karachi, Pakistan; 2 Co-Chair Research Work-Stream, World Rehabilitation Alliance, WHO, Geneva, Switzerland; 3 Johns Hopkins International Injury Research Unit, Health Systems Program, Department of International Health, Johns Hopkins Bloomberg School of Public Health, Baltimore, MD United States of America; 4 Center On Commercial Determinants of Health, Milken Institute School of Public Health, George Washington University, Washington DC, United States of America; 5 Department of Noncommunicable Diseases, Rehabilitation and Disability, World Health Organization, Geneva, Switzerland; 6 NIHR Global Health Research Unit on Global Surgery, Institute of Applied Health Research, University of Birmingham, Birmingham, B15 2TH United Kingdom; 7 Département Chiropratique, Université du Québec à Trois-Rivières, Trois-Rivières, QC Canada; 8 School of Physical and Occupational Therapy, Faculty of Medicine and Health Sciences, McGill University, Montreal, Canada; 9 College of Rehabilitation Sciences, University of Manitoba, Manitoba, Canada; 10 Department of Neurology, Christian Medical College and Hospital, Ludhiana, India; 11 Manipal Academy of Higher Education, Manipal, India; 12 School for Public Health and Primary Care, Department of Rehabilitation Medicine Maastricht The Netherlands; Adelante, Center of expertise in rehabilitation and audiology, Hoensbroek, The Netherlands; 13 Alliance for Health Policy and Systems Research, WHO, Geneva, Switzerland; 14 The Hopkins Centre and School of Health Sciences and Social Work, Griffith University, Brisbane, QLD Australia; 15 Institute for Disability and Rehabilitation Research and Faculty of Health Sciences, Ontario Tech University, Ontario, Canada; 16 Division of Physical Medicine and Rehabilitation Neuromuscular Skeletal (NMS) and Acquired Brain Injury Program (ABI) GF Strong Rehabilitation Centre, University of British Columbia, Vancouver, Canada; 17 Physical Medicine and Rehabilitation Department, Hospital Nacional Edgardo Rebagliati Martins, EsSalud, Lima, Peru; 18 Department of Biomedical, Surgical and Dental Sciences, University “La Statale”, Milan, Italy; 19 IRCCS Istituto Ortopedico Galeazzi, Milan, Italy; 20 Departments of Physical Medicine and Rehabilitation, and Physiology, University of Puerto Rico School of Medicine, PO Box 365067, San Juan, Puerto Rico; 21 Department of Social Work, Education and Community Wellbeing, Northumbria University, Newcastle Upon Tyne, United Kingdom; 22 Research Task Force, Indian Federation of Neuro Rehabilitation (IFNR), Mumbai, India; 23 Trustee/Council Member-International Affairs & Co-Vice Chair, Royal College of Occupational Therapists (RCOT), London, United Kingdom

**Keywords:** Health systems, Health policy and systems research, Rehabilitation, Disability, Universal health coverage, Sustainable Development Goals, Rehabilitation 2030, Low- and middle-income countries, World Health Organization

## Abstract

Despite recognized need and reasonable demand, health systems and rehabilitation communities keep working in silos, independently with minimal recognition to the issues of those who require rehabilitation services. Consolidated effort by health systems and rehabilitation parties, recognizing the value, power and promise of each other, is a need of the hour to address this growing issue of public health importance. In this paper, the importance and the need for integration of rehabilitation into health system is emphasized. The efforts being made to integrate rehabilitation into health systems and the potential challenges in integration of these efforts were discussed. Finally, the strategies and benefits of integrating rehabilitation in health systems worldwide is proposed. Health policy and systems research (HPSR) brings a number of assets that may assist in addressing the obstacles discussed above to universal coverage of rehabilitation. It seeks to understand and improve how societies organize themselves to achieve collective health goals; considers links between health systems and social determinants of health; and how different actors interact in policy and implementation processes. This multidisciplinary lens is essential for evidence and learning that might overcome the obstacles to the provision of rehabilitation services, including integration into health systems. Health systems around the world can no longer afford to ignore rehabilitation needs of their populations and the World Health Assembly (WHA) resolution marked a global call to this effect. Therefore, national governments and global health community must invest in setting a priority research agenda and promote the integration of rehabilitation into health systems. The context-specific, need-based and policy-relevant knowledge about this must be made available globally, especially in low- and middle-income countries. This could help integrate and implement rehabilitation in health systems of countries worldwide and also help achieve the targets of Rehabilitation 2030, universal health coverage and Sustainable Development Goals.

## Introduction

The concept of health as a state of physical, mental and social wellbeing and not merely the absence of disease or infirmity has been in existence for decades [1]. Though tremendous global collaborative efforts and resources have been invested to promote this concept, reducing the impact of diseases on functioning remains an increasing challenge [2]. This is significant because ‘functioning’, be it active or passive, is now recognized globally and strongly supported by the WHO as the third indicator of health [3]. Rehabilitation is a key strategy to optimize functioning, that is, the restoration of individuals experiencing disease and disability, to their fullest physical, mental and social wellbeing and functioning [4]. Unfortunately, rehabilitation has remained a neglected aspect of the health systems and policies worldwide [5–7]. The epidemiological, demographic and economic changes worldwide have been considered to be major contributing factors impacting health and functioning, and in turn the delivery of rehabilitation [8]. The use of the health policy and systems research (HPSR) framework that reflects systems thinking and recognizes the plurality of health systems along with their institutional dynamics was applied to our perspectives here [9], as this can help provide a better understanding of the social determinants underpinning these factors, their interactions and the impact on health and functioning [9, 10].

## Recognition and importance of rehabilitation over the years

Restoring individuals and communities experiencing chronic health conditions and disability to their best possible functioning capacity and enabling them to lead independent and productive lives can be a huge challenge [11]. This is especially pertinent due to the growing burden of disability and the substantial unmet need for rehabilitation worldwide [12]. There is a significant recognition about the important role of rehabilitation to decrease the impact of health conditions by donor agencies [7]. However, this approach remains largely unrecognized by several low- and middle-income countries (LMICs) that have not actively embraced rehabilitation within their health systems [7, 12]. Lack of political will and reluctance to health systems strengthening, particularly integrating rehabilitation within the health systems in LMICs, have been one of the several reasons for lack of implementation of rehabilitation into these national health systems and is a challenge that needs multiple approaches, including HPSR [7–12].

Global evidence on rehabilitation reveals that 2.41 billion people have health conditions that would benefit from rehabilitation contributing to 310 million years lived with disability [13]. Furthermore, a recent review revealed that the substantial unmet rehabilitation need exceeds the provision of rehabilitation services [14]. Much of the burden of disease as well as the unmet need for rehabilitation is in LMICs, where numerous barriers exist, such as availability and affordability of rehabilitation services [12]. Lack of awareness and knowledge about the benefits of rehabilitation among service users make it difficult to access rehabilitation services [12, 14]. A trend analysis of the rehabilitation needs of a group of major emerging economies, Brazil, Russia, India, China and South Africa (BRICS) showed that the needs have increased in both absolute and relative values from 1990 to 2017 [15]. Importantly, this trend analysis also showed that each of the BRICS nations had their own determinants and distribution of the problem and the risks are very different from one another [15, 16].

In high-income countries (HICs), the embedment of the rehabilitation approach within the health systems is currently insufficient to face the challenges ahead [10]. Its positioning as ‘after-care’ in most HICs healthcare systems does not facilitate the healthcare transition needed from a biomedical to a biopsychosocial approach [12]. Efforts to strengthen rehabilitation should be directed towards supporting the health system as a whole and integrating rehabilitation into all levels of healthcare [14]. This highlights the importance of conducting context-specific assessments of rehabilitation requirements in various settings to develop effective and appropriate strategies for those requiring rehabilitation services [13]. For example, substantial needs related to information about managing disability was identified in some countries as an important unmet need as opposed to access to therapeutic rehabilitation services amongst those experiencing the consequences of health conditions after stroke and their caregivers [16].

The importance and demand for high-quality rehabilitation services became strikingly evident during the coronavirus disease 2019 (COVID-19) pandemic because it was amongst the health services most severely disrupted by the pandemic. At the same time, COVID-19 actually increased rehabilitation needs, both for patients who were critically unwell with the disease, and for those who continue to experience the long-term consequences of their illness [17–19]. This situation implies the need to use the lens of the health systems approach to recognize and gain deeper insights on the complexities and dynamics of actors and institutions within the system.

Motivation behind writing this paper is that despite recognized need and reasonable demand, health systems and rehabilitation communities keep working in silos, independently without realizing that pains and problems of those who need rehabilitation services will not be solved until and unless there is a considered and consolidated effort by both parties and recognizing the value, power and promise of each other. Therefore, this paper is: (1) emphasizing the importance and need of integration of rehabilitation into health system; (2) describing the efforts being made to integrate rehabilitation into health systems and what could be the potential challenges in integration of these efforts; and, finally, (3) making a case that the use and application of HPSR can help us understand what is needed to do this successfully across countries.

## Current efforts to integrate rehabilitation into health systems

The political mandate for integrating rehabilitation into health systems was finally set into motion by the World Health Assembly (WHA) Resolution WHA76.6 on Strengthening Rehabilitation in Health Systems in May 2023 [20]. The resolution not only provides a soft law mandate for countries, but more importantly, the moral impetus and normative guidance to make integration of rehabilitation a reality at the country level. The moral impetus arises from the unanimous commitment of WHO Member States to take action to scale up rehabilitation. In addition, the resolution creates the normative vision of where rehabilitation services should be provided, how and for whom they should be made available [20].

The resolution resulted from three key factors: conceptual clarity about rehabilitation, its ultimate purpose and how to achieve this in health systems; stakeholder cohesion achieved through the Rehabilitation 2030 initiative; and the commitment of rehabilitation champions [21]. Conceptually, rehabilitation is, first and foremost, a health strategy, the primary aim of which is to optimize everyday functioning. Rehabilitation assists individuals – children, adults or older people – in achieving optimal independence in daily activities; enabling participation in education, employment and leisure; and fulfilling life roles such as caregiving for family members [7]. This is accomplished by collaborating with the individual and their family to address underlying health conditions and their symptoms, adapting the environment to accommodate their needs better, utilizing assistive tools, providing education to reinforce self-management and modifying tasks to ensure safer and more independent performance [21]. Collectively, these strategies support individuals in overcoming challenges related to cognition, vision, hearing, communication, eating or mobility.

Integrating rehabilitation services into health systems ensures equity so that everyone with a health condition who needs rehabilitation receives quality services to optimize and maintain their functioning in everyday life [20]. This is fundamental to the WHA resolution’s vision since in many countries and settings around the world, not even 50% of those who could benefit from rehabilitation have access to quality rehabilitation services [21]. The potential path to achieving health equity is the one proposed by WHO, and that is universal health coverage (UHC) using a health system strengthening approach. In practice, for the vision of UHC to include rehabilitation, all components of the health system – governance and leadership, workforce, health information systems, financing, and medicines and assistive products – must be strengthened so that high-quality and integrated rehabilitation services can be made available to everyone who needs them. Integration of rehabilitation services requires that rehabilitation is not only available at the tertiary level of care but, most importantly, at secondary and primary levels [19–21].

Rehabilitation 2030 has been the vehicle for achieving the second key factor leading to the WHA Resolution, namely stakeholder cohesion [15]. Rehabilitation 2030 was launched in 2017 to draw attention to the profound unmet need for rehabilitation worldwide [12]. Since then, rehabilitation stakeholders – professional organizations, academics and researchers, bilateral organizations, ministries of health and others – have been working together to produce evidence, develop normative tools for health system strengthening and support countries [19]. Two examples of such evidence are global estimates of the need for rehabilitation worldwide [14] and the theme issue of the WHO Bulletin on advancing rehabilitation through health policy and system research [21]. These are significant pieces of evidence because they firmly position rehabilitation in the public health, the health policy and systems research agendas. The Rehabilitation 2030 stakeholders have also developed normative tools to support countries to strengthen each of the components of the health systems to successfully integrate rehabilitation. An overview of these normative tools can be found on the WHO web resources [22]. These tools are being implemented in the context of Rehabilitation 2030 in more than 40 countries [23].

The third key factor resulting in the WHA Resolution was the commitment of rehabilitation champions. These champions range from prominent individuals, such as the actor Emilia Clarke and rehabilitation professional advocates at the country level, to WHO collaborating centres and other institutions, such as the National Center for Medical Rehabilitation Research at the National Institutes of Health in the United States, professional organizations and ministries of health. As requested by the resolution, all of these champions are now utilizing the World Rehabilitation Alliance as a platform for advocacy to continue raising awareness of the need to strengthen rehabilitation in health systems [24].

## Challenges in integration of rehabilitation

First, the global efforts mentioned earlier have started to help reform health systems for rehabilitation worldwide [2]. However, integrating rehabilitation within the health systems and primary healthcare (PHC) has been a significant challenge thus far, especially in LMICs. Important reasons include the lack of awareness of the importance of rehabilitation in the health systems and the lack of in-country assistance to integrate rehabilitation within the health systems and PHC in many LMICs [25]. Despite the current efforts of getting rehabilitation on the global agenda and development of guidelines documents, contextualizing rehabilitation in the health systems has been a challenging task. Integrating rehabilitation in HSPR can help to understand the strategies to achieve the global agendas such as UHC, SDG and the Rehab 2030, such as the need for reorienting the health systems to integrate rehabilitation and recognizing that to be a sustainable solution for achieving these global agendas will require tools, guidelines, implementation learning, HPSR and more [21, 26].

Rehabilitation’s neglect in health systems, particularly in LMICs, originates from misconceptions – first and foremost that rehabilitation is a disability-specific service needed exclusively by persons with disabilities [27]. In addition, in HICs rehabilitation is at times considered to be a luxurious and complex intervention requiring a wide range of professional multi-disciplinary inputs for which there is little evidence for effectiveness [28]. Additionally, classical medical research may not help, because evaluation of complex rehabilitation interventions require a different model, typical of complex interventions and HPSR [29], while the purely biological approach to medical research may fall short [30]. Given the complex ways in which health systems function in every country, integrating a complex intervention such as rehabilitation within the health systems has been an immense challenge. The lack of main-streaming rehabilitation within every pillar of the health systems such as policies, services, financing, supplies, information management and governance has been a reason for this challenge [26].

Second, in many countries the ministries of health (MOH) and ministries for social care or welfare (MOSC) are distinct organizations, and often they do not function together [31]. In general, MOH looks at the health aspects of the general population, while the MOSC is responsible for groups with specific needs, such as persons with disabilities. In countries in which rehabilitation is still considered a disability-specific service, MOSC administer rehabilitation leading to lack of proper integration of rehabilitation [32]. Thus, the WHO, in the context of Rehabilitation 2030, is promoting and supporting countries to integrate rehabilitation into health systems and as part of universal health coverage.

The lack of inter-ministerial and inter-sectoral coordination has hampered the effectiveness of health systems, resulting in the slow integration of rehabilitation [33]. The absence of local political will for such integration and inter-sectoral action has been a predisposing factor for this continued challenge in many LMICs [34]. In HICs, integration has been primarily approached from a service-delivery perspective. These integrated services include comprehensive rehabilitation organized through institutional facilities; however, the effectiveness of these integrated services in the communities remains a challenge [35].

The allocation of financial resources for health and social care is often based on an assumption that the needs of the people requiring health and those who need social care are very similar [36]. However, this is not often the case, as their needs constantly change, especially regarding rehabilitation. This situation does not recognize the quantum of rehabilitation needs among community-dwellers with health conditions and poor functioning where outreach services are sparse and still evolving. Investing and financing integrated health and social care services has been a complex challenge even within institutional facilities providing rehabilitation and more difficult for community outreach services [36].

Third, the structure of the health and social care systems and the complexities of their functioning to meet the rehabilitation needs have provoked various strategic approaches, valued particularly during the pandemic [37]. It became evident that rehabilitation cannot be the work of only the MOSC to achieve the integration of rehabilitation in UHC and especially to address the underpinnings of social determinants of health (SDH) [26]. There is also a need for having a multi-dimensional and trans-disciplinary approach to enhance inter-sectoral actions for integrating rehabilitation in health systems and primary care worldwide [26].

To deliver high-value rehabilitation care, countries must have the capacity to do so efficiently [38]. Unfortunately, there is an acute shortage of rehabilitation professionals in LMICs and systems of care. Given the absence of rehabilitation within health systems, the available workforce usually migrates out of LMICs. These professionals often move to HICs for a more conducive work environment and where organized pathways for rehabilitation exist [39]. Development of effective policies and pathways for enhancing the Whole of Government (WOG) approach could be a potential strategy to increase equitable access to rehabilitation and address the unmet needs in the context of LMICs [39].

Developing inclusive evidence-based policies for rehabilitation presents a complex challenge [40]. Investment in development, evaluation and implementation of rehabilitation policy interventions will be critical to achieving the Rehabilitation 2030 agenda [41, 42]. Policies require evidence for scalable rehabilitation interventions; and generating evidence for rehabilitation interventions especially using HPSR could help policymakers in rehabilitation decide on the best policy for implementation [43].

## Role of health policy and systems research for improving access to rehabilitation

Health policy and systems research is now a mature field, with several committed communities who can help strengthen research aiming to improve rehabilitation efforts. The Alliance for Health Policy and Systems Research (the Alliance), a hosted partnership at the WHO, has for 25 years built the field, provided support and guides for training and research itself and worked with other partners to generate impactful research [44]. Health Systems Global (HSG), the international society of health policy and systems researchers, now has 2000+ members in 125 countries (www.healthsystemsglobal.org). There are established centres for health policy and systems research now in a number of LMIC institutions. The broader implementation science agenda also has strong centres of excellence and support across a number of academic centres, development partners and funders in both HICs and LMICs. The lessons of success and failures of HPSR for other conditions, with a number of impactful experiences, can be usefully applied to new areas including rehabilitation.

HPSR brings a number of assets that may assist in addressing the obstacles discussed above to universal coverage of rehabilitation. It seeks to understand and improve how societies organize themselves to achieve collective health goals; considers links between health systems and social determinants of health; and how different actors interact in policy and implementation processes [45]. It is by its nature multi-disciplinary, drawing from diverse fields including economics, sociology, anthropology, political science, public health and epidemiology. This multi-disciplinary lens is essential for evidence and learning that might overcome the obstacles to the provision of rehabilitation services, including integration into health systems. Understanding governance across sectors, drawing from political science, is required to bridge the silos of social care and inclusive health service delivery. Sociological approaches, going beyond social epidemiology, are necessary to engage with the marginalization and lack of trust in the health sector experienced by many people who require rehabilitation, who often face structural discrimination within the health sector and broader society, or who are ill-served by an overly medicalized model of health [11]. The provision of rehabilitation also challenges dominant vertical models of healthcare on the basis of acute, single-episode delivery of services more suited to infectious diseases. Developing and implementing effective models of integrated chronic care service delivery – also required for the management of a range of conditions such as cardiovascular disease, cancer, diabetes, human immunodeficiency virus (HIV), tuberculosis and mental health – requires an understanding of organizational theory and change, as well as embedded implementation research, a subset of health policy and systems research [46–48].

Another asset of HPSR towards improving access to rehabilitation is its commitment to co-creating knowledge between policymakers, researchers, implementers and communities, also an important underpinning of implementation research [49, 50]. It is crucial that the needs, perspectives and challenges of communities that require rehabilitation and those delivering rehabilitation services are central to defining what is being researched for the generation of appropriate knowledge that can assist in overcoming the barriers to rehabilitation. HPSR marks itself out by its commitment to active engagement with those experiencing policy and implementation challenges and questioning the dominance of academic researchers in the process of research. The evidence that will shift resources, policy and practice on rehabilitation to better meet people’s rights and needs is unlikely to be best communicated through journal articles or academic seminars, but will instead be owned and communicated in the language and settings of policymakers, implementers and users. However, health and medical journals can play an important role in catalysing the argument, on the basis of science, for the political, policy and development communities.

## Conclusions

Health systems around the world can no longer afford to ignore rehabilitation needs of their populations and the WHA resolution marked a global call to this effect. However, implementing this vision for UHC to encompass and build out rehabilitation services will require more than a global call – it will need policy support, financing and actual implementation of models on the ground. It needs a movement that is decentralized to recognize and value rehabilitation within the health systems. For this to happen, this paper proposes the use of HPSR and associated learning pathways to help countries realize this goal. Therefore, national governments and the global health community must invest not just their resources, but also their intellectual expertise in setting a priority research agenda and in further building the field of integration of rehabilitation into health systems. This effort must consider the association of not just experts, but also frontline health and rehabilitation workforce. This would enhance a context-specific, need-based and policy-relevant knowledge available in the countries, especially in LMICs. This would also potentially result in an inclusive formulation and implementation of rehabilitation policies within the health system. The above is essential to realize the commitments and agenda developed and proposed by the global health community, as well as also to make improved policy and management decisions at the country (and sub-national) level to strengthen rehabilitation services within the national health systems worldwide.

## Data Availability

No datasets were generated or analysed during the current study.

## Abbreviations

WHO: World Health Organization
HPSR: Health policy and systems research
LMICs: Low- and middle-income countries
HICs: High-income countries
BRICS: Brazil, Russia, India, China and South Africa
PwD: Persons with disabilities
UHC: Universal health coverage
WHA: World Health Assembly
SDGs: Sustainable Development Goals
PHC: Primary healthcare
ICF: International Classification of Functioning Disability and Health
MoH: Ministry of Health
MoSC: Ministry of Social Care
WoG: Whole of Government
SDH: Social determinants of health
