# Correction: Role and promise of health policy and systems research in integrating rehabilitation into the health systems

**DOI:** 10.1186/s12961-024-01244-1

**Published:** 2024-11-08

**Authors:** Abdul Ghafar, Abdulgafoor M. Bachani, Adnan A. Hyder, Alarcos Cieza, Aneel Bhangu, André Bussières, Diana C. Sanchez-Ramirez, Dorcas B. C. Gandhi, Jeanine Verbunt, Kumanan Rasanathan, Louise Gustafsson, Pierre Côté, Rajiv Reebye, Roger De la Cerna-Luna, Stefano Negrini, Walter R. Frontera, Sureshkumar Kamalakannan

**Affiliations:** 1 Department of Community Health Sciences, Aga Khan University, Karachi, Pakistan; 2 Co-Chair Research Work-Stream, World Rehabilitation Alliance, WHO, Geneva, Switzerland; 3 Johns Hopkins International Injury Research Unit, Health Systems Program, Department of International Health, Johns Hopkins Bloomberg School of Public Health, Baltimore, MD USA; 4 Center On Commercial Determinants of Health, Milken Institute School of Public Health, George Washington University, Washington, DC USA; 5 Department of Noncommunicable Diseases, Rehabilitation and Disability, World Health Organization, Geneva, Switzerland; 6 NIHR Global Health Research Unit on Global Surgery, Institute of Applied Health Research, University of Birmingham, Birmingham, B15 2 UK; 7 Département Chiropratique, Université du Québec à Trois-Rivières, Trois-Rivières, QC Canada; 8 School of Physical and Occupational Therapy, Faculty of Medicine and Health Sciences, McGill University, Montreal, Canada; 9 College of Rehabilitation Sciences, University of Manitoba, Manitoba, Canada; 10 Department of Neurology, Christian Medical College and Hospital, Ludhiana, India; 11 Manipal Academy of Higher Education, Manipal, India; 12 School for Public Health and Primary Care, Department of Rehabilitation Medicine Maastricht The Netherlands; Adelante, Center of Expertise in Rehabilitation and Audiology, Hoensbroek, The Netherlands; 13 Alliance for Health Policy and Systems Research, WHO, Geneva, Switzerland; 14 The Hopkins Centre and School of Health Sciences and Social Work, Grifth University, Brisbane, QLD Australia; 15 Institute for Disability and Rehabilitation Research and Faculty of Health Sciences, Ontario Tech University, Ontario, Canada; 16 Division of Physical Medicine and Rehabilitation Neuromuscular Skeletal (NMS) and Acquired Brain Injury Program (ABI) GF Strong Rehabilitation Centre, University of British Columbia, Vancouver, Canada; 17 Physical Medicine and Rehabilitation Department, Hospital Nacional Edgardo Rebagliati Martins, EsSalud, Lima, Peru; 18 Department of Biomedical, Surgical and Dental Sciences, University “La Statale”, Milan, Italy; 19 IRCCS Istituto Ortopedico Galeazzi, Milan, Italy; 20 Departments of Physical Medicine and Rehabilitation, and Physiology, University of Puerto Rico School of Medicine, PO Box 365067, San Juan, Puerto Rico; 21 Department of Social Work, Education and Community Wellbeing, Northumbria University, Newcastle Upon Tyne, UK; 22 Research Task Force, Indian Federation of Neuro Rehabilitation (IFNR), Mumbai, India; 23 Trustee/Council Member-International Affairs & Co-Vice Chair, Royal College of Occupational Therapists (RCOT), London, UK

**Correction: Health Research Policy and Systems (2024) 22:143** 10.1186/s12961-024-01235-2

Following publication of the original article [1] it was reported that there was an error in the affiliations of Pierre Côté and Rajiv Reebye.

Pierre Côté was mistakenly assigned affiliation 5 instead of 15, and Rajiv Reebye was mistakenly assigned affiliation 15 instead of their missing affiliation, now affiliation 16.

The incorrect affiliations were:

Pierre Côté^5^, Rajiv Reebye^15^

^5^Department of Noncommunicable Diseases, Rehabilitation and Disability, World Health Organization, Geneva, Switzerland.

^15^Institute for Disability and Rehabilitation Research and Faculty of Health Sciences, Ontario Tech University, Ontario, Canada.

The correct affiliations are:

Pierre Côté^15^, Rajiv Reebye^16^

^15^Institute for Disability and Rehabilitation Research and Faculty of Health Sciences, Ontario Tech University, Ontario, Canada

^16^Division of Physical Medicine and Rehabilitation Neuromuscular Skeletal (NMS) and Acquired Brain Injury Program (ABI) GF Strong Rehabilitation Centre, University of British Columbia, Vancouver, Canada

As a consequence of the renumbering due to Rajiv Reebye’s missing affiliation the original affiliations 16–22 have been renumbered to 17–23.

The original article has been updated.
